# Short-Term Changes in Weather Conditions and the Risk of Acute Coronary Syndrome Hospitalization with and without ST-Segment Elevation: A Focus on Vulnerable Subgroups

**DOI:** 10.3390/medicina60030454

**Published:** 2024-03-09

**Authors:** Andreea-Alexandra Rus, Mihai-Andrei Lazăr, Romeo Negrea, Alina-Ramona Cozlac, Cristina Văcărescu, Raluca Şoşdean, Silvia-Ana Luca, Dan Gaiţă, Cristian Mornoş

**Affiliations:** 1 Department of Cardiology, “Victor Babes” University of Medicine and Pharmacy, 2 Eftimie Murgu Square, 300041 Timisoara, Romania; andreea.rus@umft.ro (A.-A.R.); nicoramo_alina@yahoo.com (A.-R.C.); cristina.vacarescu@umft.ro (C.V.); sosdean.raluca@umft.ro (R.Ş.); silvia.luca@umft.ro (S.-A.L.); dgaita@cardiologie.ro (D.G.); mornos.cristian@umft.ro (C.M.); 2 Research Center of the Institute of Cardiovascular Diseases Timisoara, 13A Gheorghe Adam Street, 300310 Timisoara, Romania; 3 Institute of Cardiovascular Diseases Timisoara, 13A Gheorghe Adam Street, 300310 Timisoara, Romania; 4 Department of Mathematics, Politehnica University, 300006 Timisoara, Romania; romeo.negrea@upt.ro

**Keywords:** acute coronary syndrome, climate changes, meteorological factors, older adult patients, vulnerable subgroups, environmental risk factors

## Abstract

*Background and Objectives*: Acute coronary syndrome (ACS), a prevalent global cardiovascular disease and leading cause of mortality, is significantly correlated with meteorological factors. This study aims to analyze the impact of short-term changes in meteorological factors on the risk of ACS, both with and without ST-segment elevation, and to identify vulnerable subgroups. *Materials and Methods*: Daily ACS admissions and meteorological variables were collected from October 2016 to December 2021. A generalized linear model (GLM) with a Poisson distribution was employed to examine how short-term fluctuations in meteorological parameters influence ACS hospitalizations. Subgroup analyses were conducted to identify the populations most vulnerable to climate change. *Results*: Multiple regression analyses showed that short-term fluctuations in atmospheric pressure (≥10 mbar) and air temperature (≥5 °C) seven days prior increased the number of ACS hospitalizations by 58.7% (RR: 1.587; 95% CI: 1.501–1.679) and 55.2% (RR: 1.552; 95% CI: 1.465–1.644), respectively, notably impacting ST-segment elevation myocardial infarctions (STEMIs). The least pronounced association was observed between the daily count of ACS and the variation in relative air humidity (≥20%), resulting in an 18.4% (RR: 1.184; 95% CI: 1.091–1.286) increase in the risk of hospitalization. Subgroup analysis revealed an increased susceptibility among men and older adults to short-term variations in weather parameters. *Conclusions*: The findings indicate that short-term changes in weather conditions are associated with an increased risk of ACS hospitalizations, particularly STEMIs. Male and older adult patients exhibit heightened susceptibility to variations in climatic factors. Developing effective preventive strategies is imperative to alleviate the adverse consequences of these environmental risk factors.

## 1. Introduction

Acute coronary syndrome (ACS) continues to be the leading cause of death globally. Cardiovascular diseases account for over half of the mortality rate in Romania, a figure nearly twice as high as the reported data in the rest of Europe. An increase in coronary artery disease has been observed since the early 20th century [1,2]. The first evidence of the interaction between weather and ACS incidence dates back to the early 1930s [3,4]. Over the years, many epidemiological studies have observed an increased incidence of acute myocardial infarctions (AMI) during colder seasons in very different geographical areas, such as Brazil, Germany, Australia, Japan, Portugal, France, and the USA [5,6,7,8,9,10,11]. The physiopathological mechanism by which climate changes determine the increase in the number of AMI hospitalizations remains uncertain. It has been observed that exposure to low atmospheric temperatures causes an increase in the serum level of catecholamines, fibrinogen, and platelets, as well as an increase in blood pressure and heart rate [12,13]. In the context of global warming in recent years, the hot months seem to be associated more and more with the increase in the number of hospitalizations due to ACS [14,15]. It has been observed that thermal stress causes an increase in plasma cholesterol levels, blood viscosity, as well as the number of platelets and red cells [13,16].

In addition to the influence of seasonal variations, epidemiological studies have also analyzed the impact of different meteorological parameters (such as air temperature, relative humidity, atmospheric pressure, precipitation, and wind speed) on the number of hospitalizations due to ACS [10,17,18,19,20]. “V”, “U”, or “J” shaped associations between atmospheric pressure, air temperature, and AMI incidence have been described [10,17,20]. While the effect of variations in meteorological parameters on AMI admissions has been extensively documented, few studies have examined the relationship between these meteorological variables and the type of ACS (ST-segment elevation myocardial infarction—STEMI vs. non-ST-segment elevation acute coronary syndrome—NSTE-ACS), especially in vulnerable populations.

This study aims to analyze the impact of short-term changes in meteorological factors on the risk of ACS, with and without ST-segment elevation, and to identify vulnerable subgroups. The results can enhance our understanding of the association between meteorological phenomena and ACS incidence, aiding in the development of plans to mitigate the health impacts of climate change and reduce public health program costs.

## 2. Materials and Methods

### 2.1. Study Population

This epidemiological observational analysis evaluates the statistical associations between the number of hospitalizations due to ACS and the variation in meteorological factors seven days before the acute coronary event. Patients with a diagnosis of ACS undergoing coronary angiography between October 2016 and December 2021 were selected from the register of the University Hospital in Timisoara, Romania. To be eligible, we included in the current study only patients who fulfilled the following criteria: 1. Age 18 years or more. 2. Main diagnosis at admission of STEMI (chest pain of at least 20 min; persistent ST-segment elevation of at least 1 mm; increase and/or decrease in the high-sensitivity troponin I serum level) and NSTE-ACS (precordial pain lasting 15–20 min or an episode of angina pectoris lasting more than 20 min; persistent ST segment depression of at least 1 mm; increase and/or decrease in the high-sensitivity troponin I serum level—non-ST-segment elevation myocardial infarction/NSTEMI or normal serum values of biomarkers of myocardial necrosis—unstable angina/UA). Patients who did not live in the geographic area of interest for at least seven days before the onset of the acute coronary event, as well as those diagnosed with chronic coronary syndrome (CCS), were excluded from the data collection.

Cardiovascular risk factors (smoking, dyslipidemia, hypertension, diabetes, and heredity), time of onset of symptoms, various clinical parameters at admission, thrombolytic treatment and interventional management, or other treatment received during hospitalization were also collected.

The current study complies with the Declaration of Helsinki and was approved by the Scientific Research Ethics Commission of the “Victor Babes” University of Medicine and Pharmacy in Timisoara, Romania (No. 25/31.05.2021). Written informed consent was obtained from all patients included in this study.

### 2.2. Meteorological Data

The geographical study area has a temperate continental climate, with more moderate air temperatures and more precipitation. In Romania, the maximum average annual temperatures were recorded between 22–24 °C in the summer months and between −3 and −5 °C in the winter period, with an average annual precipitation of 637 mm [21]. The University Hospital of Timisoara receives cardiovascular emergencies from the southwestern region of Romania, a large geographical area with a permanent population of 1,706,965 in 2023, according to the estimate of the National Institute of Statistics [22].

The climatic variables were provided by the National Meteorological Administration of Romania. The data from this paper were collected from the nearest weather station where the patient lived for at least seven days before the onset of ACS. The following meteorological variables were included for the analysis: atmospheric temperature (minimum, maximum, and average; °C), atmospheric pressure (minimum, maximum, and average; mbar), relative air humidity (average; %), precipitation (average; mm/24 h), wind speed (maximum, average; m/s), sunshine duration (average; h/day), and cloud cover (average; h/day). Meteorological factors were recorded as values from the day of hospitalization (day 0) until seven days before the onset of the acute coronary event (day 7). Later, the variation in each parameter was determined for the seven days preceding the onset of ACS. Meteorological variables were gathered from five regions of the country, each characterized by distinct meteorological conditions, resulting in a cumulative dataset spanning a total of 3475 days

### 2.3. Statistical Analysis

Numerical variables were presented as mean ± SD (standard deviation) and were compared using the Independent Samples *t*-test. Categorical variables were expressed as percentages and were compared using Pearson’s chi-squared test.

Considering the Poisson-type distribution of daily ACS hospitalizations, we used a generalized linear model (GLM) with Poisson distribution to explore the impact of short-term changes in meteorological variables on ACS admissions. Therefore, an overall analysis and subgroup analysis (including male, female, older adult, diabetic, and hypertensive patients) were performed according to the type of ACS (STEMI vs. NSTE-ACS) to identify the subpopulations most vulnerable to climate change. The results were expressed as relative risk (RR) along with 95% confidence intervals (CI). The statistical analysis employed R software (version 4.2.0) with a significance threshold established at *p* < 0.05, while graphical representations were generated using Excel Version 2019.

## 3. Results

This research included a total of 5300 patients hospitalized for ACS. Among them, 66.1% (3504 individuals) were admitted for STEMI, while 33.9% (1796 patients) were admitted for NSTE-ACS (NSTEMI and UA). The distribution between genders indicated a higher prevalence among men (72.2%) compared to women (27.8%). The average age of the hospitalized individuals was 62.1 ± 11.5 years. The main therapeutic approach was primary percutaneous coronary intervention (PCI), accounting for 85.6% of the overall hospitalized patients. Within the subgroup diagnosed with STEMI, only 16.7% (884 individuals) underwent thrombolysis before admission. Among the older adult population (≥65 years), the prevalence was 41.8% for STEMI and 48% for NSTE-ACS (*p* = 0.0036). Notably, 46.7% of smokers presented with STEMI upon admission, whereas patients with diabetes (33.4% vs. 23.6%, *p* < 0.001) and hypertension (87.2% vs. 64.5%, *p* < 0.001) were more prominently represented in the NSTE-ACS group. Gender differences did not reach statistical significance between the two ACS subtypes. Table 1 provides a comprehensive overview of the fundamental characteristics of patients diagnosed with ACS.

**Figure 1:**
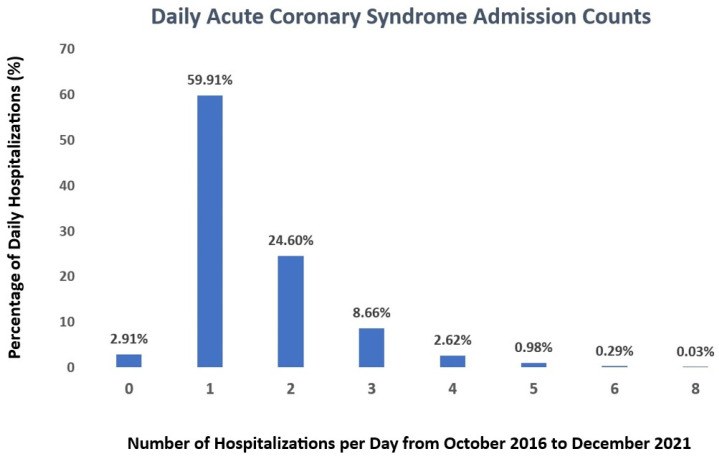
Daily counts of ACS admissions from October 2016 to December 2021. A single hospitalization occurred on approximately 59.91% of days, 33.26% of all days recorded 2-3 hospitalizations per day, while only 3.92% saw four or more admissions. In 2.91% of instances, there were no recorded hospitalizations. All data are presented in percentage terms. The values correspond to the following: 0—zero hospitalizations per day; 1—one hospitalization per day; 2—two hospitalizations per day; 3—three hospitalizations per day; 4—four hospitalizations per day; 5—five hospitalizations per day; 6—six hospitalizations per day; 8—eight hospitalizations per day.

The average daily admission rate during this period was 2.1 ± 1.2. A single hospitalization occurred on approximately 59.91% of days, while only 3.92% saw four or more admissions. In 2.91% of instances, there were no recorded hospitalizations. The majority of hospitalizations occurred during the winter season (December to February), constituting 27% of the total. Analysis of hospitalizations by the day of the week revealed the highest frequency on Monday, accounting for 16.5%, while Sunday recorded the lowest frequency at 10.3%. Figure 1 depicts the daily counts of ACS admissions from October 2016 to December 2021.

During the study period, the mean daily atmospheric temperature was 11.8 ± 8.7 °C, atmospheric pressure stood at 999.8 ± 11.1 mbar, relative humidity averaged 73.6 ± 14.1%, wind speed was recorded at 1.9 ± 1.0 m/s, duration of sunshine amounted to 5.7 ± 4.2 h per day, cloudiness averaged 5.4 ± 3.3 h per day, and the average precipitation amounted to 1.7 ± 4.4 mm in 24 h. Table 2 provides a comprehensive overview of the statistical measures, such as mean, standard deviations (SD), minimum and maximum values, and selected percentiles, relevant to the meteorological factors investigated in this paper.

**Figure 2:**
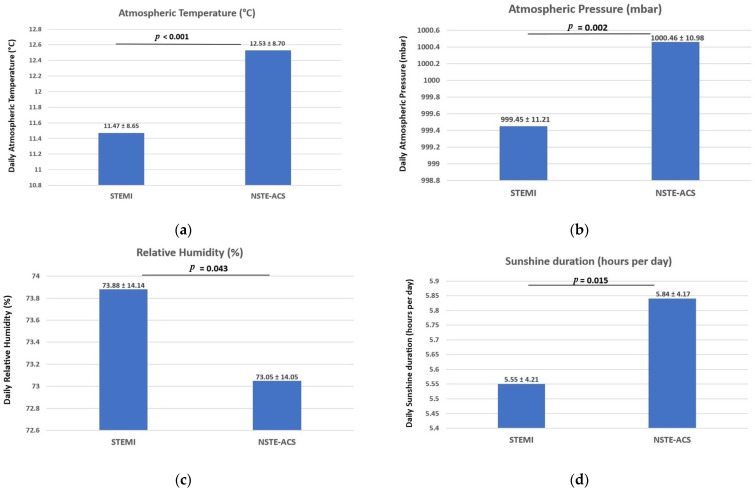
Meteorological variables based on subtypes of acute coronary syndrome. An increased incidence of hospitalizations due to STEMI could be observed on days with lower air temperatures (**a**) and atmospheric pressure (**b**), higher air humidity (**c**), and less sunny days (**d**) compared to the daily number of NSTE-ACS admissions. Data were expressed as mean ± SD. A *p*-value less than 0.05 indicates a statistically significant association; STEMI = ST-segment Elevation Myocardial Infarction; NSTE-ACS = Non-ST-segment Elevation Acute Coronary Syndrome; SD = Standard Deviation.

Figure 2 and Supplementary Table S1 examine the mean daily values of meteorological parameters according to ACS subtypes (STEMI vs. NSTE-ACS).

An increased incidence of hospitalizations due to STEMI could be observed on days with lower air temperatures (Figure 2a) and atmospheric pressure (Figure 2b), higher air humidity (Figure 2c), and less sunny days (Figure 2d) compared to the daily number of NSTE-ACS admissions.

Table 3 shows the connections between the daily hospitalization occurrences related to ACS and one-unit changes in meteorological variables during the seven days preceding the onset of the acute coronary event.

**Figure 3:**
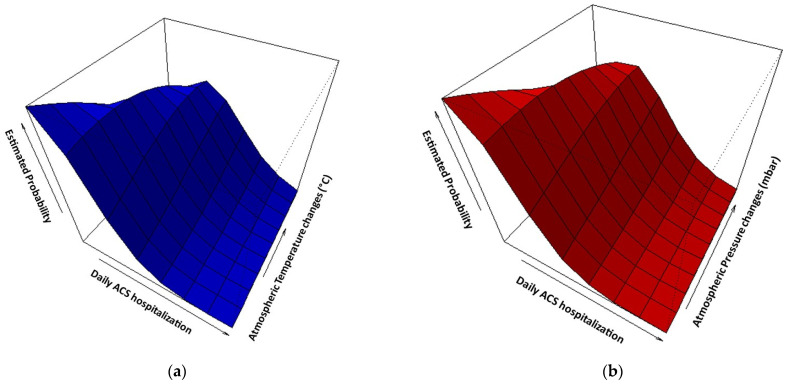

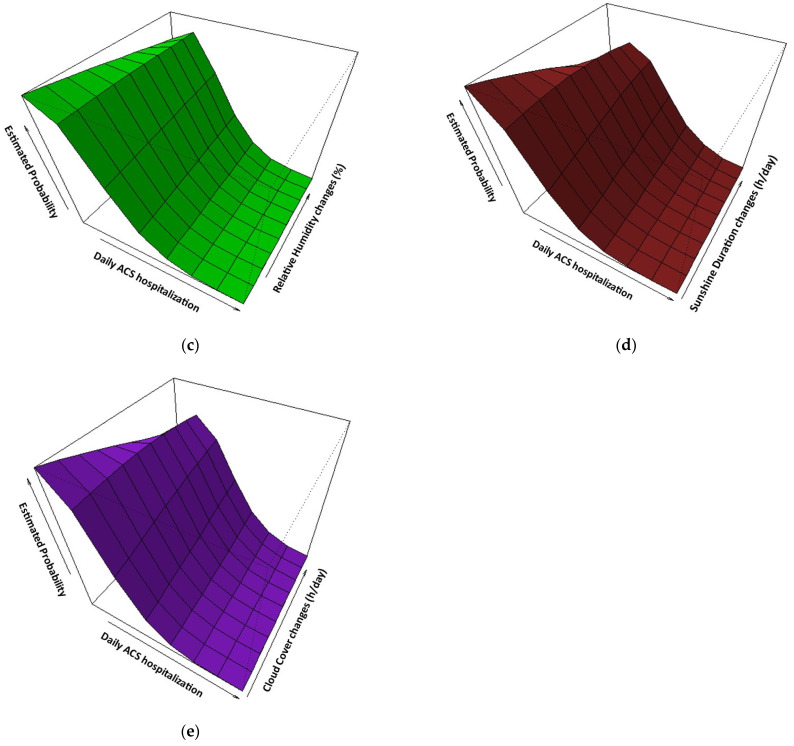
Three-dimensional visualization of the correlation between acute coronary syndrome admissions and meteorological variable fluctuations with a 7-day lag. There is a higher likelihood of increased daily ACS hospitalizations when there is a greater variation in air temperature (**a**), atmospheric pressure (**b**), relative air humidity (**c**), sunshine duration (**d**), and cloudiness (**e**) seven days before the acute coronary event. ACS—Acute Coronary Syndrome.

Multiple regression analyses indicated that a one-unit change in air temperature, atmospheric pressure, duration of sunshine, and cloudiness is associated with statistically significant increases in the daily number of hospitalizations, elevating the risk of ACS by 5.9% (RR: 1.059; 95% CI: 1.048–1.070), 2.4% (RR: 1.024; 95% CI: 1.019–1.028), 4.7% (RR: 1.047; 95% CI: 1.032–1.062), and 3.9% (RR: 1.039; 95% CI: 1.023–1.056), respectively. Moreover, a modest yet statistically significant relationship was observed between relative humidity and the daily number of admissions, leading to a 0.5% (RR: 1.005; 95% CI: 1.001–1.008) increase in the risk of acute cardiac events. However, no significant associations were identified between the daily occurrences of AMI and variations in wind speed and precipitation. After excluding non-statistically significant variables, Figure 3a–e illustrate that there is a higher likelihood of increased daily admissions when there is greater variation in meteorological factors seven days prior.

**Figure 4:**
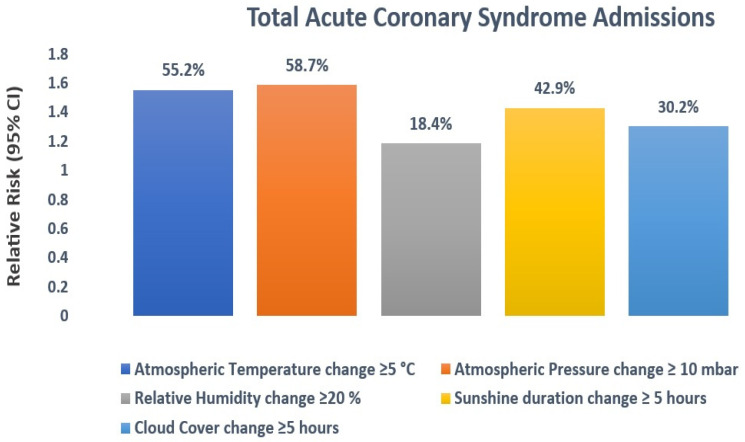
Estimating the relative risk for the entire population with acute coronary syndrome in relation to weather parameter changes, with a 7-day lag. Only meteorological variables with a value of *p* < 0.05 were graphically represented. An RR value > 1 signifies an elevated risk, and <1 indicates a diminished risk.

As evidenced in the additional Figure S1–S5, the highest probability of having two daily hospitalizations occurred when, seven days preceding the cardiac event, there were fluctuations of 5 °C in air temperature, 10 mbar in atmospheric pressure, 20% in relative humidity, and 5 h in the duration of sunshine and cloudiness. These specific variations were chosen as threshold values for calculating the risk associated with having two or more daily admissions due to ACS. A statistically significant relationship was identified between the short-term changes in air temperature (≥5 °C) and atmospheric pressure (≥10 mbar) and the daily count of hospitalizations, resulting in a respective 55.2% (RR: 1.552; 95% CI: 1.465–1.644) and 58.7% (RR: 1.587; 95% CI: 1.501–1.679) increase in the risk of ACS. Furthermore, the incidence of AMI rose by 42.9% (RR: 1.429; 95% CI: 1.342–1.522) and 30.2% (RR: 1.302; 95% CI: 1.213–1.397) when the duration of sunshine and cloudiness varied by ≥5 h over the preceding week. The least pronounced association was observed between the daily count of ACS and the variation in relative air humidity (≥20%), resulting in an 18.4% (RR: 1.184; 95% CI: 1.091–1.286) increase in the risk of hospitalization. Figure 4 depicts the impact of meteorological factor variations on ACS admissions with a 7-day lag.

**Figure 5:**
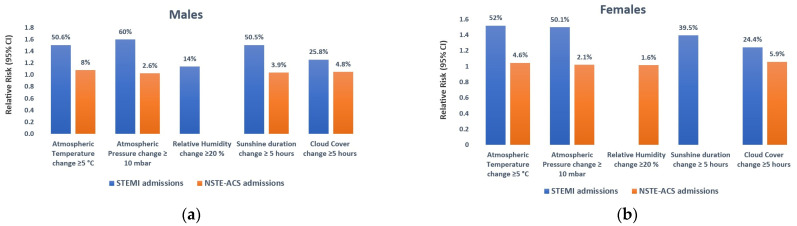
Estimating the relative risk for the gender subgroup in cases of STEMI and NSTE-ACS in association with changes in meteorological parameters, with a 7-day lag. Male patients demonstrated a heightened sensitivity to changes in atmospheric pressure, resulting in a notable 60% increase in the risk of STEMI hospitalizations, while variations in relative humidity had a comparatively minimal impact, contributing to a modest 14% increase in the occurrence of AMIs (**a**). Females demonstrated greater vulnerability to fluctuations in air temperature, resulting in a 52% increase in the risk of AMI admissions, with no impact of relative humidity on the number of STEMI hospitalizations (**b**). Only meteorological variables with a value of *p* < 0.05 were graphically represented. An RR value > 1 signifies an elevated risk, and <1 indicates a diminished risk. STEMI—ST-segment Elevation Myocardial Infarction; NSTE-ACS—Non-ST-segment Elevation Acute Coronary Syndrome; AMI — Acute Myocardial Infarction; RR—Relative Risk; CI—Confidence Interval.

We also investigated the influence of short-term weather parameter changes on ACS subtypes (STEMI vs. NSTE-ACS) across gender and vulnerable populations. The detailed findings of the subgroup analysis are presented in Supplementary Table S2. Short-term climate changes consistently demonstrated a substantially heightened risk of STEMI hospitalization compared to NSTE-ACS across all investigated subgroups. The variation in meteorological factors occurring seven days prior was associated with a greater increased risk of STEMI among men compared to females. Male patients were much more sensitive to atmospheric pressure changes, leading to a 60% (RR: 1.600; 95% CI: 1.478–1.732) increase in the risk of STEMI hospitalizations. Simultaneously, females demonstrated greater vulnerability to fluctuations in air temperature, resulting in a 52% (RR: 1.520; 95% CI: 1.333–1.733) increase in the risk of AMI admissions (Figure 5a,b).

**Figure 6:**
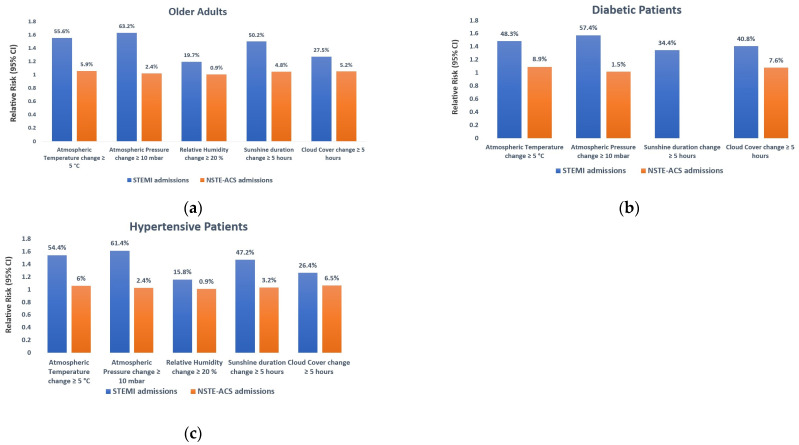
Estimating the relative risk for the age subgroup and patients with cardiovascular risk factors in cases of STEMI and NSTE-ACS in association with changes in meteorological parameters, with a 7-day lag. In the susceptible subpopulation, alterations in atmospheric pressure exerted the most pronounced influence, leading to a 63.2% elevation in the risk of STEMI hospitalization among older adults (**a**), a 57.4% increase in diabetics (**b**), and a 61.4% rise in hypertensive patients (**c**). The fluctuations in relative humidity had a more modest impact, resulting in a 19.7% increase in daily hospitalizations among older adults (**a**) and a 15.8% rise among hypertensive patients (**c**), with no statistically significant association observed in diabetics (**b**). Only meteorological variables with a value of *p* < 0.05 were graphically represented. A RR value > 1 signifies an elevated risk, and <1 indicates a diminished risk. STEMI—ST-segment Elevation Myocardial Infarction; NSTE-ACS—Non-ST-segment Elevation Acute Coronary Syndrome; RR—Relative Risk; CI—Confidence Interval.

Regarding the vulnerable subpopulation, alterations in meteorological conditions have resulted in a more pronounced disease burden among older adults (≥65 years) compared to individuals with diabetes and hypertension. A variation in atmospheric pressure of ≥10 mbar elevated the risk of STEMI hospitalization by 63.2% (RR: 1.632; 95% CI: 1.473–1.808) among older adults, 57.4% (RR: 1.574; 95% CI: 1.373–1.805) in diabetics, and 61.4% (RR: 1.614; 95% CI: 1.489–1.750) in hypertensive patients. Similarly, changes in atmospheric temperature were associated with an increase in the daily count of STEMI admissions by 55.6% (RR: 1.556; 95% CI: 1.402–1.728) in older adults, 48.3% (RR: 1.483; 95% CI: 1.287–1.708) in diabetics, and 54.4% (RR: 1.544; 95% CI: 1.419–1.680) in hypertensive patients. The variation in relative humidity had a modest impact on the risk of STEMI, resulting in a 19.7% (RR: 1.197; 95% CI: 1.031–1.388) increase in daily hospitalizations among older adults and a 15.8% (RR: 1.158; 95% CI: 1.029–1.304) increase among hypertensive patients, with no significant association observed in diabetics. All of these particulars are depicted in Figure 6a–c.

## 4. Discussion

The results obtained from the present study reveal a robust association between short-term fluctuations in climate factors, including air temperature, atmospheric pressure, relative humidity, duration of sunshine, and cloudiness, and admissions for ACS in the Eastern European region. The estimated relative risk exhibited variations based on gender, age, and other cardiovascular risk factors. To our knowledge, this is the first epidemiological observational study from our geographical region that identifies a correlation between changes in environmental variables occurring seven days prior and the daily frequency of ACS hospitalizations, encompassing both STEMI and NSTE-ACS cases, particularly within vulnerable subgroups.

Many prior epidemiological investigations have examined the correlation between the occurrence of acute coronary disease and the average levels of meteorological factors, typically assessing values from the day of admission or within a few days or weeks preceding hospitalization [10,17,18,19,20,23,24,25]. Nevertheless, a limited number of studies have delved into the impact of meteorological parameter variations on the risk of STEMI and NSTE-ACS in vulnerable subcategories, including those with major cardiovascular risk factors [26,27,28,29,30,31].

The findings from our study indicate a heightened occurrence of daily ACS cases during the winter period, similar to most previous research [5,6,7,8,9,10,11]. Notably, a study conducted in Japan revealed an increased incidence of AMI cases and mortality rates during both winter and spring, while data from a French study demonstrated elevated occurrences of STEMI in the winter and autumn months [8,25]. Limited epidemiological data have reported an increased number of hospitalizations due to AMI in the hot season [14,15].

While the exact mechanisms remain not fully known and comprehended, various pathophysiological explanations have been proposed to elucidate why exposure to either cold or heat can trigger acute coronary disease. Exposure to both low and high temperatures has been associated with an augmentation in platelet and red cell counts, an elevation in fibrinogen concentration, and increased blood viscosity, ultimately leading to heightened thrombogenicity [12,13,16].

The examination of ACS subtypes revealed a heightened occurrence of STEMI cases and a decreased number of admissions for NSTE-ACS on days characterized by lower air temperatures and atmospheric pressures, higher relative humidity, and fewer sunny days. Consistent with our findings, Wang et al. demonstrated a significant relationship between AMI occurrence and days featuring low atmospheric pressure and air temperatures, particularly when the average daily temperature fell below 10 °C [23]. In alignment with these observations, a comprehensive national study reported an elevated risk of AMI occurrence on days with low temperatures and atmospheric pressure, high wind speeds, and a shorter duration of sunshine [32]. On the other hand, other data suggested a significant correlation between the frequent occurrence of AMI and decreased air temperature, along with heightened atmospheric pressure, low humidity, and prolonged sunshine duration [33]. Reports from a study in China revealed an uptick in the number of STEMI hospitalizations during periods of high apparent temperatures and an increased incidence of NSTEMI on days with low apparent temperatures [34].

In the context of the entire analyzed population, our study reveals that the most significant association exists between the daily frequency of ACS hospitalizations and a change in atmospheric pressure of ≥10 mbar occurring seven days before admission, leading to a 58.7% increase in the risk of coronary events. Similar observations were reported by Versaci et al. in their study conducted in Italy, where the rate of AMI was higher during spring, particularly on days with pronounced variations in atmospheric pressure [35]. Other authors, in their research, showed an elevated incidence of AMI when atmospheric pressure increased rapidly within the 24 h preceding the event, while a study conducted in France highlighted a “V”-shaped relationship between atmospheric pressure and the incidence of coronary events [10,36]. Furthermore, Danet et al. reported a 12% increase in the incidence of acute heart disease for every 10 mbar drop in atmospheric pressure below 1016 mbar and an 11% increase for every 10 mbar rise above this threshold [10]. However, several studies did not find a significant relationship between the occurrence of AMI and air pressure [31,37,38].

Conflicting outcomes have been documented in studies examining the correlation between air temperature and the incidence of acute coronary diseases. Our findings reveal a 55.2% elevation in the risk of ACS when the atmospheric temperature varies by at least 5 °C in the week preceding the event. These observations are consistent with previous studies that demonstrated an increased risk of cardiovascular disease and stroke in older adults when exposed to a 3 °C fluctuation in air temperature, as well as a 14% increase in the risk of acute cardiac events associated with daily temperature changes of ≥5 °C [30,31]. Moreover, various researchers have described linear, “J”-, or “U”-shaped correlations between environmental temperature and the frequency of ACS hospitalizations [10,20,39].

A slight correlation was evident between the daily occurrence of AMI and a change in air humidity of at least 20% in the week before the event, resulting in an 18.4% increase in the risk of hospitalization. Previous studies have presented varying findings on the relationship between relative humidity and the incidence of ACS, with some reporting a positive correlation, others indicating a negative relationship, and one study finding no association between the number of hospitalizations and this meteorological parameter [31,38,40,41,42].

We identified no significant associations between ACS hospitalizations and variations in wind speed and precipitation, similar to results from previous research [33,38,43]. Conversely, conflicting reports exist, with some confirming a positive association between wind speed and the daily incidence of AMI, while others showed a negative relationship [36,44,45,46]. Notably, a study conducted in China demonstrated an increased incidence of ACS on days with abundant precipitation, contrasting with observations from research in Japan that suggested a positive correlation between low precipitation and the occurrence of STEMI [47].

The outcomes of our study showed a 42.9% and 30.2% elevation in the risk of ACS when the duration of sunshine and cloudiness varied by ≥5 h in the week preceding hospitalization. Research conducted in Sweden indicated an increased incidence of AMI on days with a short duration of sunshine, while a study in Japan reported an elevated risk on sunny days [32,33].

By examination of ACS subtypes (STEMI vs. NSTE-ACS), we demonstrated a strong association between short-term meteorological parameter changes and the daily count of hospitalizations for STEMI, in contrast to NSTE-ACS, across all patient categories. Within the gender subgroup, our results indicated a higher increase in the relative risk of STEMI in males compared to females. This suggests that men exhibit greater susceptibility to climate changes, particularly atmospheric pressure variations. Other studies have identified males as one of the most vulnerable groups, while additional epidemiological data have observed that females may be more susceptible to short-term weather changes [26,27,28,29]. In the age subgroup and among patients with cardiovascular risk factors, our findings show that the most susceptible category to climate variations was older adults, followed by hypertensive patients and, lastly, diabetics. Atmospheric pressure and air temperature changes exerted the greatest impact on STEMI admissions, while relative humidity variation demonstrated a modest effect, increasing the risk of AMI by 19.7% in older adults and 15.8% in hypertensive patients, with no impact among diabetics. Similar to our results, other epidemiological data identified older adults as the most susceptible category, while Boussoussou et al. reported an increased incidence of acute cardiovascular disease among diabetic and dyslipidemic patients and those with a history of heart disease [24,27,29,30,31]. The older adult population generally presents with multiple comorbidities associated with a compromised immune system and reduced capacity to adapt to climate changes, rendering them the most vulnerable to short-term variations in meteorological factors. Contrary to our reported findings, an epidemiological study conducted in a rural area of China showed a higher increase in the risk of hospitalizations due to cardiovascular diseases among adults compared to older adult patients, particularly in conditions of low apparent temperature [26].

The presented results can provide a better understanding of the association between meteorological phenomena and the incidence of ACS in order to develop plans to reduce the negative effects on health caused by climate change, consequently reducing the financial burden on public health programs. Enhancing our comprehension of the mechanisms by which variations in meteorological parameters elevate the risk of acute coronary events may enable the development of effective preventive strategies. Anticipated global climate change in the upcoming years is linked to diverse alterations in various weather parameters. Thorough examinations with extended study populations are needed to elucidate the complex biological mechanisms by which atmospheric parameters may contribute to the destabilization of atherosclerosis plaques, consequently influencing the onset and prognosis of coronary heart disease.

## 5. Conclusions

Weather parameters exert a notable influence on the incidence of ACS. The best predictors correlated with the hospitalization rate of ACS were the short-term fluctuations of at least 10 mmHg in atmospheric pressure and at least 5 °C in air temperature observed seven days before the acute coronary event. Hospitalizations due to STEMI exhibited greater sensitivity to fluctuations in meteorological factors compared to NSTE-ACS. This heightened susceptibility was particularly pronounced among male individuals and older adults in response to climate change.

The outcomes of this study have the potential to furnish governments with strategic measures to manage the escalating rate of hospitalizations and enhance the capacity to prevent cardiovascular diseases. This could involve the timely issuance of notices to vulnerable populations, prompting them to safeguard against extreme meteorological factor variations. The findings presented herein may carry significant implications for advancing our understanding of health impacts related to climate change and promoting a more efficient allocation of local public health resources.

### Study Limitations

This study has several limitations. The exclusion of patients who succumbed before hospital arrival or those not admitted to any hospital (outpatients) may result in an underrepresentation of the sample. Individual differences, including medical history, lifestyle, social status, as well as information about pharmacotherapy, were not incorporated into the research due to a lack of pertinent data. Weather data from fixed monitoring sites provides a generalized representation of individual exposures within the entire study population. No analyses were conducted regarding atmospheric pollutants, and no examination was undertaken to assess the interaction among distinct meteorological variables. The present study was conducted in a limited geographical area (the country’s southwestern region). We examined the correlation between temporal variations in meteorological factors over the previous seven days and ACS within the week. Multiple time frames, including daily or monthly occurrences, could have been assessed, resulting in numerous combinations. However, our decision to focus on the preceding seven days and the weekly incidence was based on its simplicity and alignment with the established data in the literature.

## Figures and Tables

**Table 1:** Patient characteristics.

	ACS (n = 5300)
Age (years)	62.1 ± 11.5
Gender	
(male)	3827 (72.2%)
(female)	1473 (27.8%)
Body mass index (kg/m^2^)	28.7 ± 5.1
Cardiovascular risk factors	
Smoking	2104 (39.7%)
Diabetes mellitus	1427 (26.9%)
Arterial hypertension	3826 (72.2%)
Hypercholesterolemia	1978 (37.3%)
Family History	188 (3.5%)
Clinical parameters at admission	
SBP (mmHg)	138.4 ± 23.6
DBP (mmHg)	81.5 ± 14.1
HR (bpm)	77.4 ± 21.0
Coronary angiography	
Single-vessel lesion	2164 (40.8%)
Two-vessel lesion	1702 (32.1%)
Three-vessel lesion	1434 (27.1%)
LVEF (%)	43.7 ± 7.9
Type of treatment	
Conservative	484 (9.1%)
Balloon angioplasty	94 (1.8%)
Angioplasty and stent placement	4537 (85.6%)
CABG	185 (3.5%)

Data were expressed as mean ± SD or number (%). ACS = Acute Coronary Syndrome; SD = Standard Deviation; SBP = Systolic Blood Pressure; DBP = Diastolic Blood Pressure; HR = Heart Rate; LVEF = Left Ventricular Ejection Fraction; CABG = Coronary Artery Bypass Grafting.

**Table 2:** Daily meteorological features.

Total Days (n = 3475)	Mean ± SD	Minimum	Percentiles			Maximum
Variables			25th	50th	75th	
Atmospheric Temperature (°C)	11.8 ± 8.7	−15.4	4.9	11.2	19.1	31.5
Atmospheric Pressure (mbar)	999.8 ± 11.1	963.3	991.3	1001.0	1007.3	1033.9
Relative Humidity (%)	73.6 ± 14.1	32	63	74	85	100
Wind Speed (m/s)	1.9 ± 1.0	0.3	1.2	1.6	2.3	8.7
Precipitation (mm/24 h)	1.7 ± 4.4	0	0	0	0.9	61.2
Sunshine duration (hours per day)	5.7 ± 4.2	0	1.4	6.0	9.3	14.4
Cloud Cover (hours per day)	5.4 ± 3.3	0	2.5	5.5	8.5	10

Meteorological variables were gathered from five regions of the country, each characterized by distinct meteorological conditions, resulting in a cumulative dataset spanning a total of 3475 days. SD = Standard Deviation.

**Table 3:** The effect of the variation in meteorological factors on the ACS admissions over 7 lag days *.

Variable	Relative Risk	95% CI	*p*-Value
Atmospheric Temperature (°C)	1.059	1.048–1.070	<0.001
Atmospheric Pressure (mbar)	1.024	1.019–1.028	<0.001
Relative Humidity (%)	1.005	1.001–1.008	0.012
Wind Speed (m/s)	1.014	0.983–1.045	0.394
Precipitation (mm/24 h)	1.004	0.999–1.008	0.112
Sunshine Duration (hours per day)	1.047	1.032–1.062	<0.001
Cloud Cover (hours per day)	1.039	1.023–1.056	<0.001

* An RR value > 1 signifies an elevated risk, and <1 indicates a diminished risk. A *p*-value less than 0.05 indicates a statistically significant association. RR—Relative Risk; CI—Confidence Interval.

## Data Availability

The data supporting this article are not available for public sharing in consideration of the privacy of the study participants. However, upon reasonable request, interested parties may obtain access to the data by contacting the corresponding author.

## Supplementary Materials

The following supporting information can be downloaded at https://www.mdpi.com/article/10.3390/medicina60030454/s1, Table S1: Mean daily values of meteorological parameters according to ACS subtypes; Figure S1: The correlation between the daily average of two ACS admissions and atmospheric temperature changes with a 7-day lag, emphasizing maximum estimated probability at 5 °C air temperature variations; Figure S2: The correlation between the daily average of two ACS admissions and atmospheric pressure changes with a 7-day lag, emphasizing maximum estimated probability at 10 mmHg air pressure variations; Figure S3: The correlation between the daily average of two ACS admissions and relative humidity changes with a 7-day lag, emphasizing maximum estimated probability between 20–25% air humidity variations; Figure S4: The correlation between the daily average of two ACS admissions and sunshine duration changes with a 7-day lag, emphasizing maximum estimated probability at 5-hour variations; Figure S5: The correlation between the daily average of two ACS admissions and cloud cover changes with a 7-day lag, emphasizing maximum estimated probability at 5-hour variations; Table S2: The effect of the variation of meteorological factors on the ACS admissions over 7 lag days in different patient subgroups.
