# Supplementary material for: Short-Term Changes in Weather Conditions and the Risk of Acute Coronary Syndrome Hospitalization with and without ST-Segment Elevation: A Focus on Vulnerable Subgroups
Source: Medicina (Kaunas). 2024 Mar 9;60(3):454. doi: 10.3390/medicina60030454 (PMC10972014; doi:10.3390/medicina60030454)
Supplement: Supplementary file 1 [file medicina-60-00454-s001.zip › medicina-2897412-supplementary.pdf]

Supplementary Materials

Table S1. Mean daily values of meteorological parameters according to acute coronary syndrome subtypes*

	**STEMI**	**NSTE-ACS**	
**Variables**	**Mean ± SD**	**Mean ± SD**	***p*-value**
Atmospheric Temperature (°C)	11.47 ± 8.65	12.53 ± 8.70	**<0.001**
Atmospheric Pressure (mbar)	999.45 ± 11.21	1000.46 ± 10.98	**0.002**
Relative Humidity (%)	73.88 ± 14.14	73.05 ± 14.05	**0.043**
Wind Speed (m/s)	1.85 ± 1.01	1.89 ± 0.97	0.176
Precipitation (mm/24h)	1.68 ± 4.48	1.60 ± 4.35	0.539
Sunshine duration (hours per day)	5.55 ± 4.21	5.84 ± 4.17	**0.015**
Cloud Cover (hours per day)	5.44 ± 3.36	5.31 ± 3.26	0.180

*A p-value less than 0.05 indicates a statistically significant association; STEMI = ST-segment Elevation Myocardial Infarction; NSTE-ACS = Non-ST-segment Elevation Acute Coronary Syndrome. SD – Standard Deviation

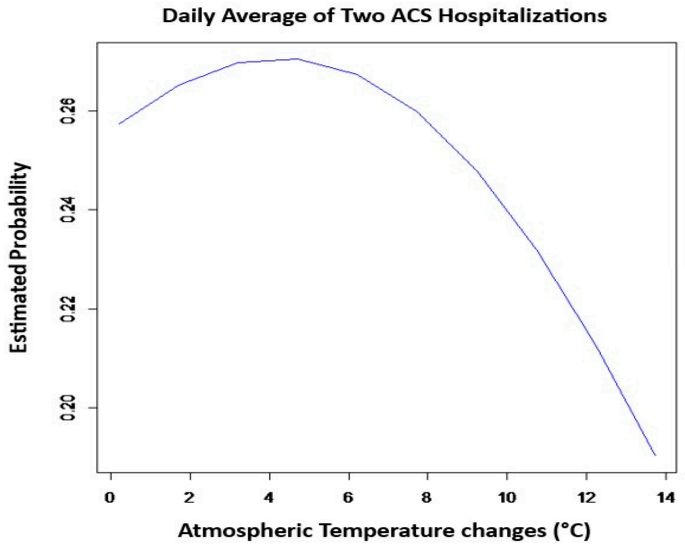

Figure S1. The correlation between the daily average of two acute coronary syndrome admissions and atmospheric temperature changes with a 7-day lag, emphasizing maximum estimated probability at 5°C air temperature variations.

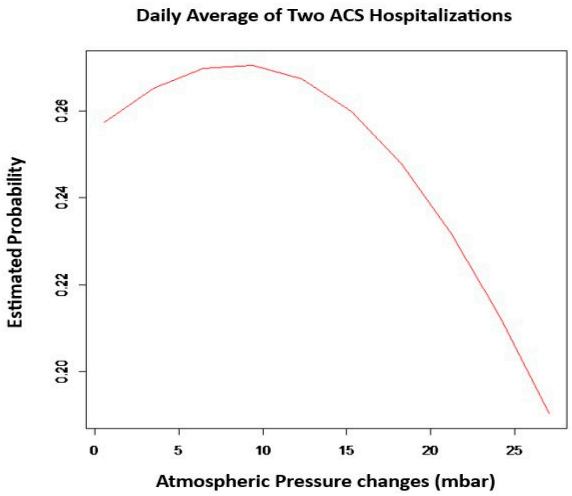

Figure S2. The correlation between the daily average of two acute coronary syndrome admissions and atmospheric pressure changes with a 7-day lag, emphasizing maximum estimated probability at 10 mmHg air pressure variations.

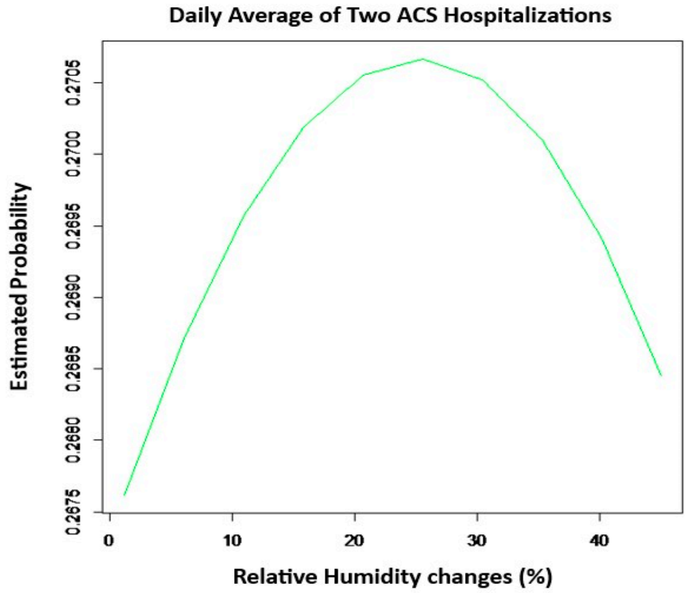

Figure S3. The correlation between the daily average of two acute coronary syndrome admissions and relative humidity changes with a 7-day lag, emphasizing maximum estimated probability between 20-25% air humidity variations.

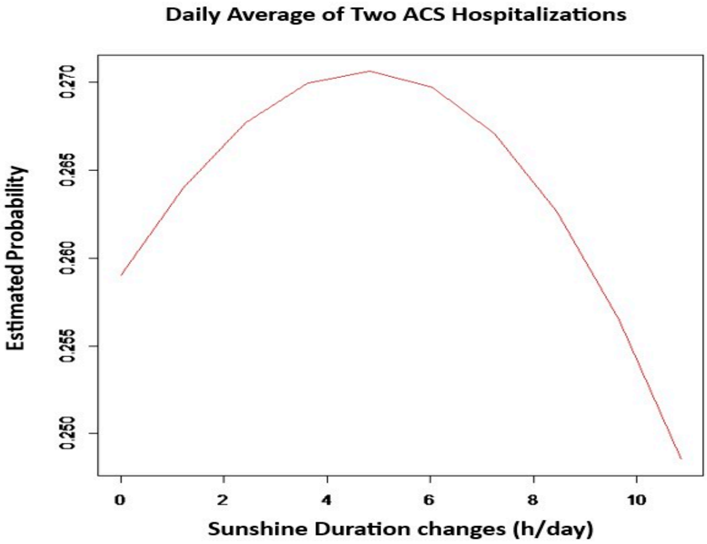

Figure S4. The correlation between the daily average of two acute coronary syndrome admissions and sunshine duration changes with a 7-day lag, emphasizing maximum estimated probability at 5-hour variations.

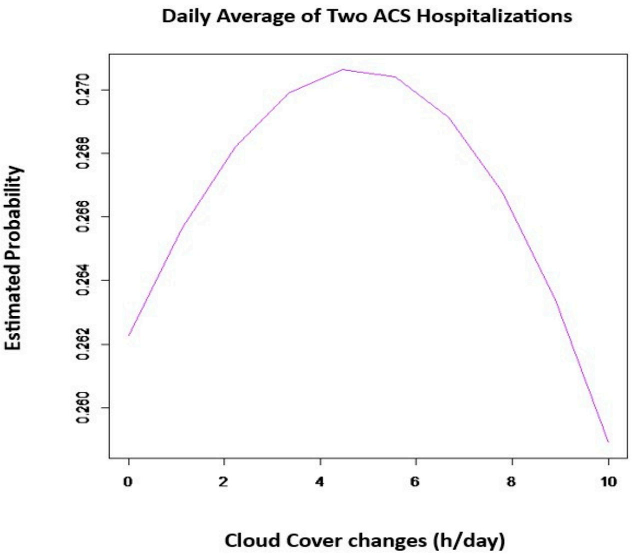

Figure S5. The correlation between the daily average of two acute coronary syndrome admissions and cloud cover changes with a 7-day lag, emphasizing maximum estimated probability at 5-hour variations.

**Table S2.** The effect of the variation of meteorological factors on the ACS admissions over 7 lag days in different patient subgroups*				
	**STEMI**		**NSTE-ACS**	
**Subgroups of patients**	**Relative Risk (95% CI)**	***P*-value**	**Relative Risk (95% CI)**	***P*-value**
**Older Adults (≥ 65 years)**				
Atmospheric Temperature change ≥5 °C	1.556 (1.402-1.728)	**<0.001**	1.059 (1.032-1.086)	**<0.001**
Atmospheric Pressure change ≥ 10 mbar	1.632 (1.473-1.808)	**<0.001**	1.024 (1.013-1.036)	**<0.001**
Relative Humidity change ≥20 %	1.197 (1.031-1.388)	**0.018**	1.009 (1.000-1.017)	**0.049**
Sunshine duration change ≥ 5 hours	1.502 (1.331-1.694)	**<0.001**	1.048 (1.011-1.017)	**0.010**
Cloud Cover change ≥5 hours	1.275 (1.115-1.458)	**<0.001**	1.052 (1.011-1.095)	**0.013**
**Females**				
Atmospheric Temperature change ≥5 °C	1.520 (1.333-1.733)	**<0.001**	1.046 (1.011-1.083)	**0.009**
Atmospheric Pressure change ≥ 10 mbar	1.501 (1.303-1.729)	**<0.001**	1.021 (1.006-1.035)	**0.005**
Relative Humidity change ≥20 %	1.186 (0.988-1.422)	0.067	1.016 (1.006-1.027)	**0.002**
Sunshine duration change ≥ 5 hours	1.395 (1.198-1.625)	**<0.001**	1.036 (0.989-1.085)	0.137
Cloud Cover change ≥5 hours	1.244 (1.049-1.476)	**0.012**	1.059 (1.005-1.115)	**0.033**
**Males**				
Atmospheric Temperature change ≥5 °C	1.506 (1.385-1.638)	**<0.001**	1.080 (1.057-1.103)	**<0.001**
Atmospheric Pressure change ≥ 10 mbar	1.600 (1.478-1.732)	**<0.001**	1.026 (1.017-1.035)	**<0.001**
Relative Humidity change ≥20 %	1.140 (1.014-1.281)	**0.029**	1.002 (0.995-1.009)	0.576
Sunshine duration change ≥ 5 hours	1.505 (1.376-1.645)	**<0.001**	1.039 (1.010-1.069)	**0.007**
Cloud Cover change ≥5 hours	1.258 (1.137-1.393)	**<0.001**	1.048 (1.016-1.081)	**0.003**
**Diabetics**				
Atmospheric Temperature change ≥5 °C	1.483 (1.287-1.708)	**<0.001**	1.089 (1.055-1.124)	**<0.001**

Atmospheric Pressure change ≥ 10 mbar	1.574 (1.373-1.805)	**<0.001**	1.015 (1.002-1.029)	**0.026**
Relative Humidity change ≥20 %	1.149 (0.944-1.399)	0.166	1.010 (1.000-1.020)	0.057
Sunshine duration change ≥ 5 hours	1.344 (1.150-1.570)	**<0.001**	1.009 (0.967-1.053)	0.682
Cloud Cover change ≥5 hours	1.408 (1.179-1.680)	**<0.001**	1.076 (1.027-1.128)	**0.002**
**Hypertensives**				
Atmospheric Temperature change ≥5 °C	1.544 (1.419-1.680)	**<0.001**	1.060 (1.039-1.082)	**<0.001**
Atmospheric Pressure change ≥ 10 mbar	1.614 (1.489-1.750)	**<0.001**	1.024 (1.016-1.033)	**<0.001**
Relative Humidity change ≥20 %	1.158 (1.029-1.304)	**0.015**	1.009 (1.002-1.015)	**0.011**
Sunshine duration change ≥ 5 hours	1.472 (1.304-1.616)	**<0.001**	1.032 (1.004-1.060)	**0.027**
Cloud Cover change ≥5 hours	1.264 (1.137-1.404)	**<0.001**	1.065 (1.033-1.099)	**<0.001**

* A RR value > 1 signifies an elevated risk, and < 1 indicates a diminished risk. A p-value less than 0.05 indicates a statistically significant association. RR – Relative Risk; CI – Confidence Interval. STEMI - ST-segment Elevation Myocardial Infarction; NSTE-ACS - Non-ST-segment Elevation Acute Coronary Syndrome;
